# Supplementary material for: Variations in climate habitability parameters and their effect on Earth's biosphere during the Phanerozoic Eon
Source: Sci Rep. 2023 Aug 4;13:12663. doi: 10.1038/s41598-023-39716-z (PMC10403619; doi:10.1038/s41598-023-39716-z)
Supplement: Supplementary file 1 — Supplementary Information. [file 41598_2023_39716_MOESM1_ESM.pdf]

Supplementary Materials

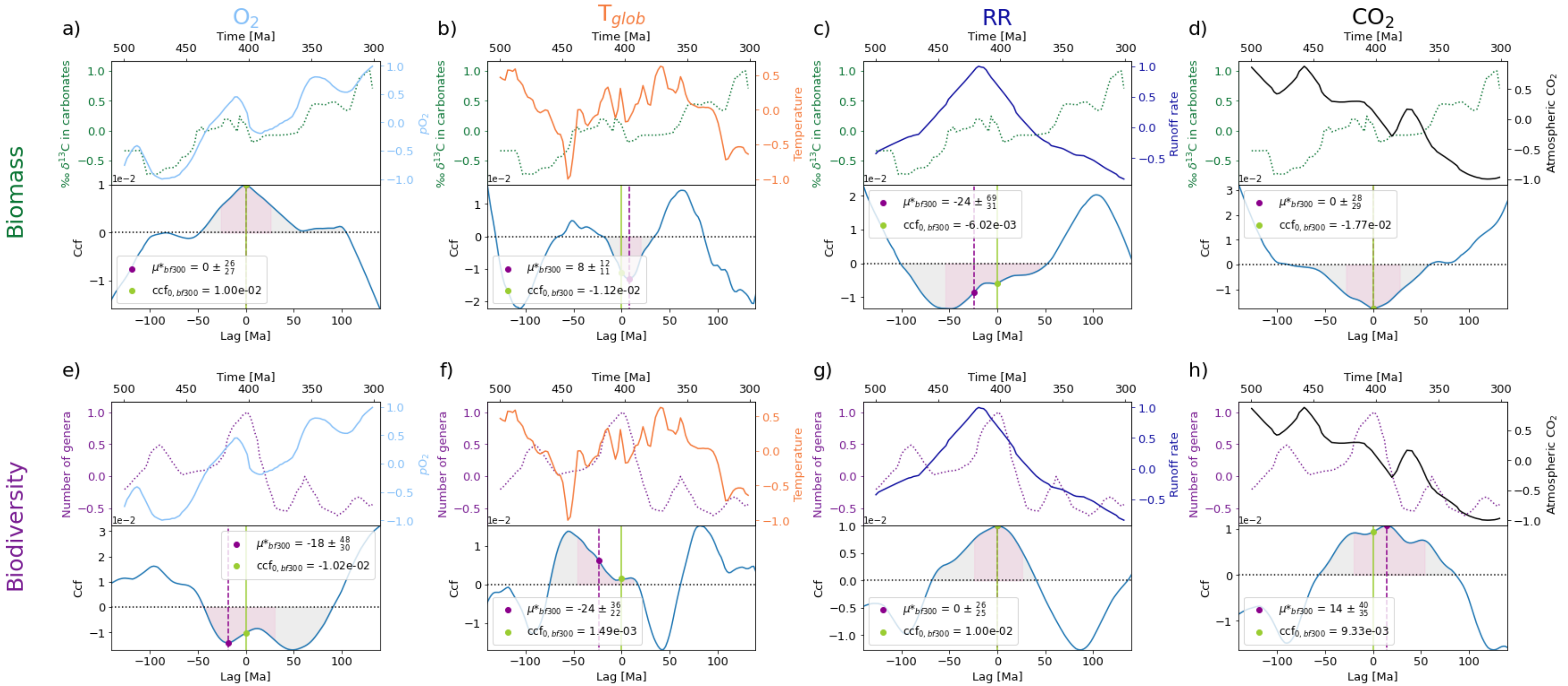

S1. Environmental and biological parameters through Earth history from 500 Ma before present until 300 Ma and their corresponding cross-correlation functions (ccf). The split at 300 Ma mimics the transition when many species started to inhabit the land rather than the oceans only[46]. The ccf provides a correlation value of the two time series at a temporal lag of 0 (ccf_0), as well as determines at which temporal lag (μ*) the time series exhibit a maximum correlation. The environmental parameters include in the upper panels of (a) through (h), respectively: mean atmospheric oxygen partial pressures pO_2, global average surface temperatures T_{glob}, runoff rates RR, and atmospheric carbon dioxide levels CO_2. The biological parameters include in the upper panels of (a) through (d) the primary productivity (in ‰ $\delta^{13}C$ in carbonates) as a proxy for biomass (dashed dark green curves) and in the upper panels of (e) through (h) the biodiversity (number of genera) of marine fauna (dotted magenta curves). Each parameter was normalized to its maximum (absolute) value after the subtraction of its corresponding mean value. This way each time series fluctuates around 0 and has the same normalized units. Bottom panels of (a) through (h): Corresponding cross-correlation function (ccf). We constrained our analysis to a ±25 Ma lag range since ever larger temporal ranges have a decreasing biogeochemical significance. The positive and negative errors with respect to μ* were calculated by determining the positive/negative temporal lags at which 34.15% of the total area between the ccf and the horizontal zero-line is encapsulated.

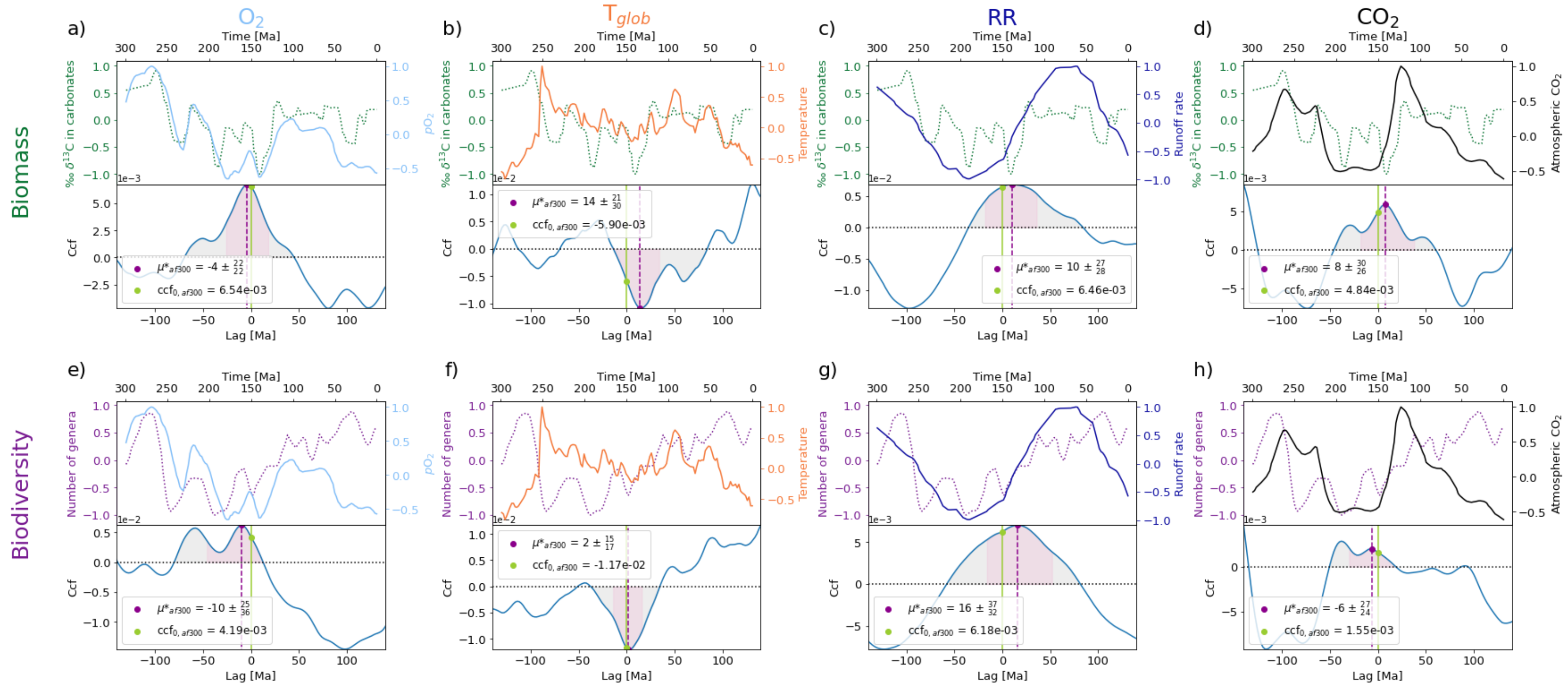

S2. Environmental and biological parameters through Earth history from 300 Ma before present until today and their corresponding cross-correlation functions (ccf). The split at 300 Ma mimics the transition when many species started to inhabit the land rather than the oceans only[46]. The ccf provides a correlation value of the two time series at a temporal lag of 0 (ccf_0), as well as determines at which temporal lag (μ*) the time series exhibit a maximum correlation. The environmental parameters include in the upper panels of (a) through (h), respectively: mean atmospheric oxygen partial pressures pO_2, global average surface temperatures T_{glob}, runoff rates RR, and atmospheric carbon dioxide levels CO_2. The biological parameters include in the upper panels of (a) through (d) the primary productivity (in ‰ $\delta^{13}C$ in carbonates) as a proxy for biomass (dashed dark green curves) and in the upper panels of (e) through (h) the biodiversity (number of genera) of marine fauna (dotted magenta curves). Each parameter was normalized to its maximum (absolute) value after the subtraction of its corresponding mean value. This way each time series fluctuates around 0 and has the same normalized units. Bottom panels of (a) through (h): Corresponding cross-correlation function (ccf). We constrained our analysis to a ±25 Ma lag range since ever larger temporal ranges have a decreasing biogeochemical significance. The positive and negative errors with respect to μ* were calculated by determining the positive/negative temporal lags at which 34.15% of the total area between the ccf and the horizontal zero-line is encapsulated.

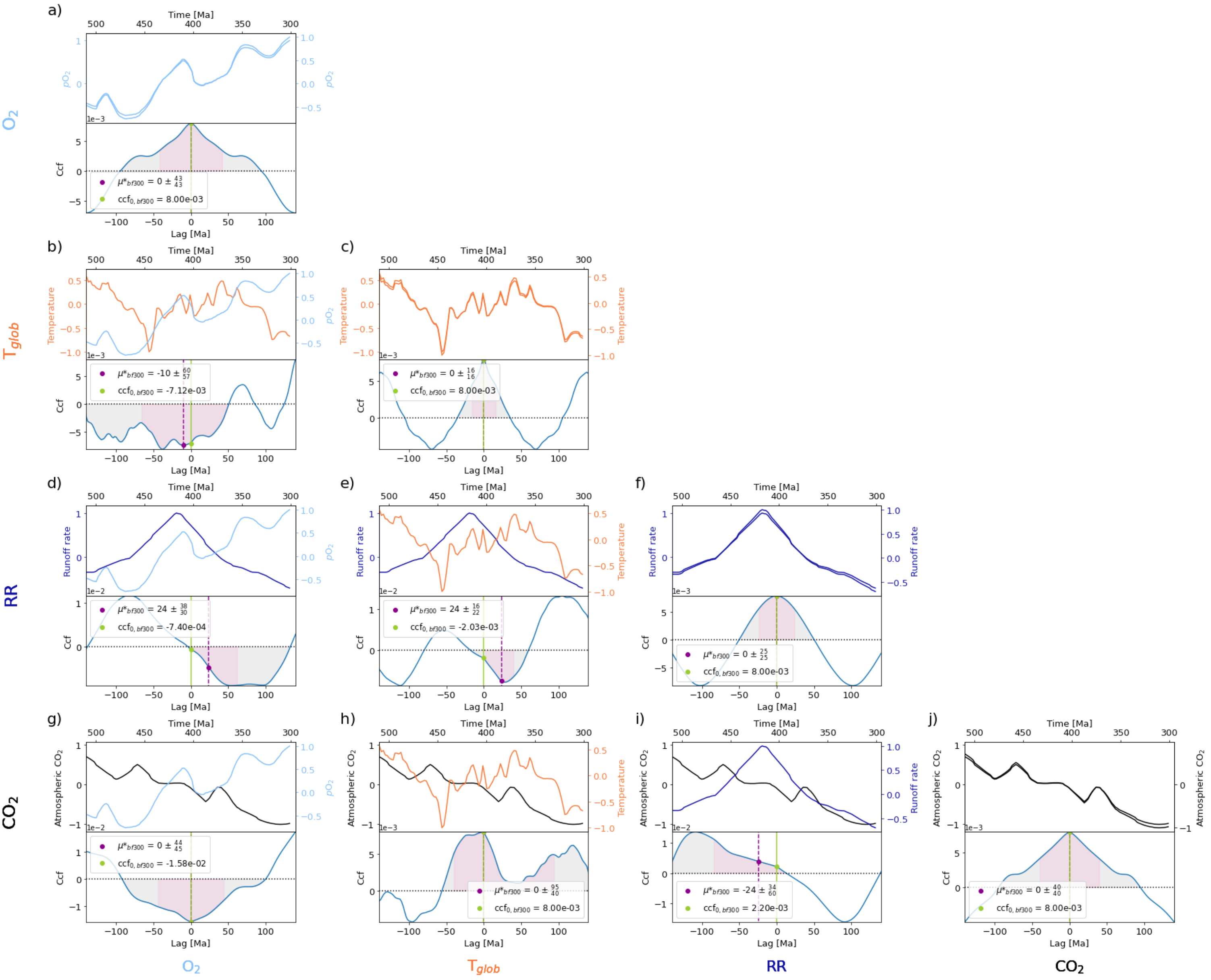

S3. Environmental parameters through Earth history from 500 Ma before present until 300 Ma ago and their corresponding cross-correlation functions (ccf). The split at 300 Ma mimics the transition when many species started to inhabit the land rather than the oceans only[46]. They include from top to bottom and left to right panel respectively: mean atmospheric oxygen partial pressures pO_2, global average surface temperatures T_{glob}, runoff rates RR, and atmospheric carbon dioxide CO_2. The auto-correlations are located on the diagonal. Other markers as in Fig. 1.

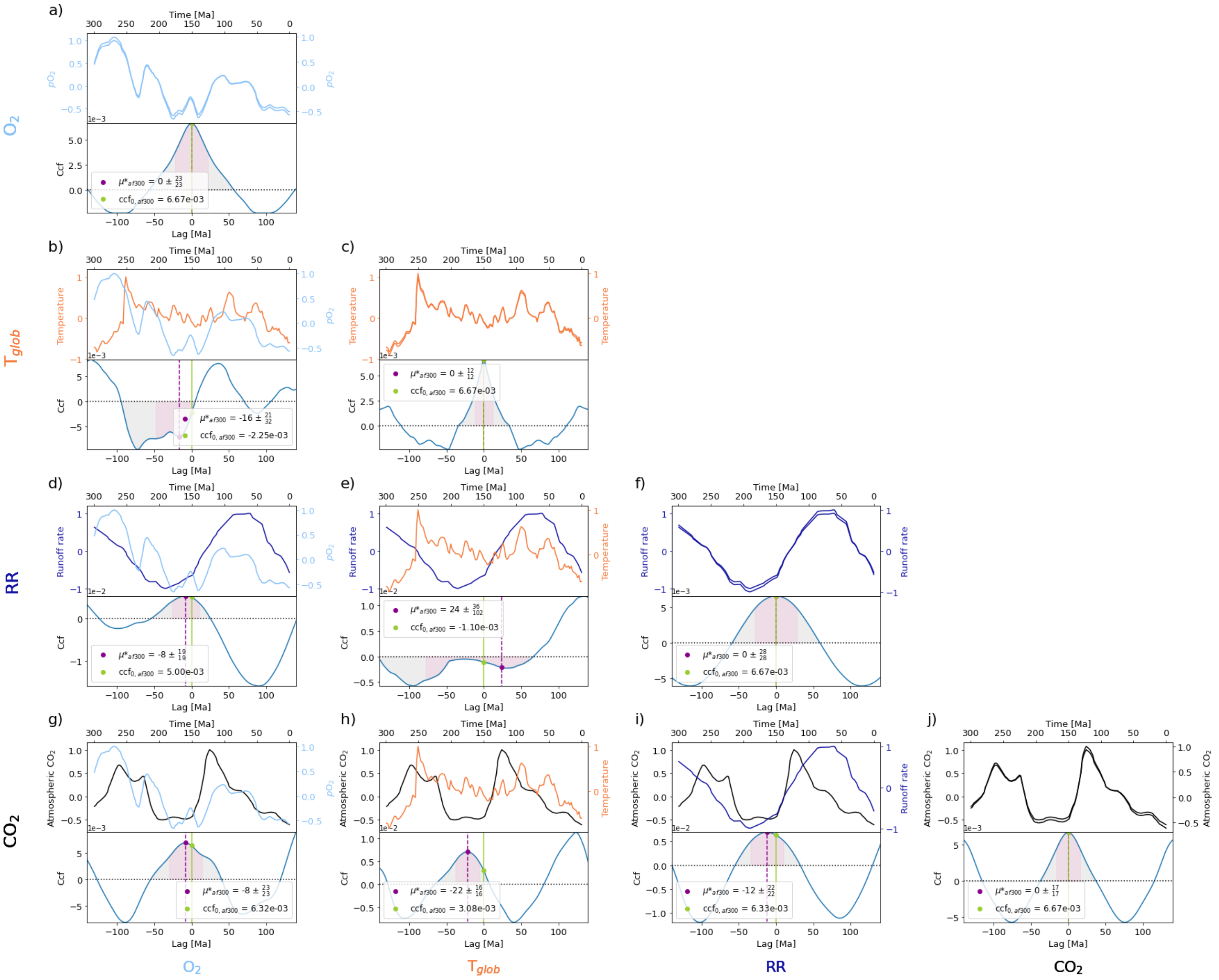

S4. Environmental parameters through Earth history from 300 Ma before present until today and their corresponding cross-correlation functions (ccf). The split at 300 Ma mimics the transition when many species started to inhabit the land rather than the oceans only[46]. They include from top to bottom and left to right panel respectively: mean atmospheric oxygen partial pressures pO_2, global average surface temperatures T_{glob}, runoff rates RR, and atmospheric carbon dioxide CO_2. The auto-correlations are located on the diagonal. Other markers as in Fig. 1.
